# Assessment of left and right ventricular functional parameters using dynamic dual-tracer [^13^N]NH3 and [^18^F]FDG PET/MRI

**DOI:** 10.1007/s12350-020-02391-y

**Published:** 2020-10-22

**Authors:** Sazan Rasul, Dietrich Beitzke, Tim Wollenweber, Ivo Rausch, Martin Lyngby Lassen, Marie Elisabeth Stelzmüller, Markus Mitterhauser, Verena Pichler, Thomas Beyer, Christian Loewe, Marcus Hacker

**Affiliations:** 1 Division of Nuclear Medicine, Department of Biomedical Imaging and Image-guided Therapy, Medical University of Vienna, Waehringer Guertel 18-20, Floor 5L, 1090 Vienna, Austria; 2 Division of Cardiovascular and Interventional Radiology, Department of Biomedical Imaging and Image-guided Therapy, Medical University of Vienna, Vienna, Austria; 3 QIMP Team, Center for Medical Physics and Biomedical Engineering, Medical University of Vienna, Vienna, Austria; 4 Artificial Intelligence in Medicine Program, Cedars-Sinai Medical Center, Los Angeles, California USA; 5 Clinical Division of General Surgery, Medical University of Vienna, Vienna, Austria; 6 Ludwig Boltzmann Institute Applied Diagnostics, Vienna, Austria

**Keywords:** Cardiac PET/MRI, dynamic PET, ^18^F-FDG, ^13^N-NH3, coronary artery disease

## Abstract

**Background:**

Cardiac positron emission tomography/magnetic resonance imaging (PET/MRI) can assess various cardiovascular diseases. In this study, we intra-individually compared right (RV) and left ventricular (LV) parameters obtained from dual-tracer PET/MRI scan.

**Methods:**

In 22 patients with coronary heart disease (69 ± 9 years) dynamic [^13^N]NH_3_ (NH_3_) and [^18^F]FDG (FDG) PET scans were acquired. The first 2 minutes were used to calculate LV and RV first-pass ejection fraction (FPEF). Additionally, LV end-systolic (LVESV) and end-diastolic (LVEDV) volume and ejection fraction (LVEF) were calculated from the early (EP) and late-myocardial phases (LP). MRI served as a reference.

**Results:**

RVFPEF and LVFPEF from FDG and NH_3_ as well as RVEF and LVEF from MRI were (28 ± 11%, 32 ± 15%), (32 ± 11%, 41 ± 14%) and (42 ± 16%, 45 ± 19%), respectively. LVESV, LVEDV and LVEF from EP FDG and NH_3_ in 8 and 16 gates were [71 (15 to 213 mL), 98 (16 to 241 mL), 32 ± 17%] and [50 (17 to 206 mL), 93 (13 to 219 mL), 36 ± 17%] as well as [60 (19 to 360 mL), 109 (56 to 384 mL), 41 ± 22%] and [54 (16 to 371 mL), 116 (57 to 431 mL), 46 ± 24%], respectively. Moreover, LVESV, LVEDV and LVEF acquired from LP FDG and NH_3_ were (85 ± 63 mL, 138 ± 63 mL, 47 ± 19%) and (79 ± 56 mL, 137 ± 63 mL, 47 ± 20%), respectively. The LVESV, LVEDV from MRI were 93 ± 66 mL and 153 ± 71 mL, respectively. Significant correlations were observed for RVFPEF and LVFPEF between FDG and MRI (*R* = .51, *P* = .01; *R* = .64, *P* = .001), respectively. LVESV, LVEDV, and LVEF revealed moderate to strong correlations to MRI when they acquired from EP FDG and NH_3_ in 16 gates (all *R* > .7, *P* = .000). Similarly, all LV parameters from LP FDG and NH_3_ correlated good to strongly positive with MRI (all *R* > .7, and *P* < .001), except EDV from NH3 weakly correlated to EDV of MRI (*R* = .54, *P* < .05). Generally, Bland-Altman plots showed good agreements between PET and MRI.

**Conclusions:**

Deriving LV and RV functional values from various phases of dynamic NH_3_ and FDG PET is feasible. These results could open a new perspective for further clinical applications of the PET examinations.

**Electronic supplementary material:**

The online version of this article (10.1007/s12350-020-02391-y) contains supplementary material, which is available to authorized users.

## Introduction

Cardiovascular diseases, especially coronary heart disease (CHD), remain the leading cause of death worldwide.^1^ Besides an optimal physical examination, various non-invasive methods such as electrocardiography, echocardiography, computed tomography angiography, cardiac magnetic resonance imaging (CMR), and cardiac positron emission tomography (PET) using different radioactive tracers are nowadays available helping accurate detection and precise determination of the underlying cardiac illnesses.

The recent development of an integrated PET/MRI imaging system could further facilitate the early diagnosis of plenty cardiovascular conditions, which might consequently improve therapy management and reduce mortality among affected patients.^2^ Due to its excellent spatial and temporal resolution and its ability to characterize myocardial composition, CMR represents a well-established imaging method in ischemic heart disease and cardiomyopathies. CMR is considered the gold standard for assessing myocardial function and can quantify myocardial scar burden in ischemic heart disease. In addition, [^13^N]NH_3_ (NH_3_) PET is often used to estimate myocardial perfusion, while the role of cardiac [^18^F]fluorodeoxyglucose (FDG) PET is mostly viability assessment, myocardial inflammation and tumor imaging. In patients with ischemic heart disease, the combination of these two tracers enables the identification of hibernating myocardium by identifying regions with perfusion-metabolism mismatch.^3^

On the other side, whole-body PET examinations using FDG are widely performed for oncological purpose like preoperative staging, characterization of suspicious tissues and lesions and response evaluation after receiving chemo- and/or immunotherapies.^4^ Although the availability of anticancer therapies has dramatically improved the survival rate of patients with different malignancies, these treatments are often associated with cardiotoxicities and subsequent serious cardiovascular adverse effects, mostly through congestive heart failure and left ventricular systolic dysfunction.^5^ Actually, due to the excellent soft tissue contrast of MRI in combination with molecular and metabolic PET data, the combined PET/MRI examination with FDG is increasingly used in oncology.^6^ Therefore, simultaneous evaluation of oncological status and cardiac function of these patients while performing a whole-body FDG PET/MRI scan might recognize patients at risk who still do not clinically demonstrate cardiotoxic effects of antineoplastic drugs.

In this context, various preclinical and human studies have demonstrated the feasibility of determining LV parameters using FDG PET and showed excellent correlations between left ventricular volume and function in static scans in humans.^7^,^8^ However, while the feasibility to derive RV and LV functional parameters from early dynamic PET scans was demonstrated in rodents,^9^ such studies with FDG PET in humans are missing or very limited.^10^,^11^

In the present study, we aimed to conduct an intra-individual comparison of the cardiac function and volume parameters of the right (RV) and left (LV) ventricle derived from different phases of dynamic FDG and NH_3_ PET scans. As integrated PET/MRI systems enable the simultaneous measurement of both PET and MRI, a simultaneous intra-individual comparison with respective CMR parameters as a reference was feasible.

## Patients and Methods

### Patients

Integrated cardiac PET/MRI (3-Tesla, Biograph mMR; Siemens Healthcare, Erlangen Germany) in head-first supine position was performed in patients with coronary heart disease and ischemic cardiomyopathy, who were referred to our department for assessing myocardial perfusion and viability with a dual-tracer NH_3_ and FDG protocol. Only proper and completely performed cardiac dual-tracer PET/MRI studies were included in this retrospective analysis. Prior to the conduction of the scan, all patients have undersigned an informed consent for the examination. The study has been approved by the Ethics Committee of the Medical University of Vienna (EK: 1832/2016).

### Protocol for Dual-Tracer PET/CMR Examination

The cardiac PET examinations for ECG-gated NH_3_ and FDG as well as the CMR examination acquired with the integrated PET/MRI followed well-established protocols that are applied for cardiac PET and CMR.^12^,^13^ All patients underwent blood glucose monitoring and received an oral 50 g glucose solution (glucoral, Germania Pharmazeutika, AUT) 1 hour before the FDG PET scan. Only diabetics with fasting blood glucose above 160 mg/dL did not receive the solution and obtained an intravenous bolus injection of a short-acting insulin followed by continuous monitoring of their blood glucose. Moreover, all patients received 250 mg of Acipimox (Olbetam®) orally 2 hours before the examination; this leads to an excellent image quality and ensures an optimal myocardial FDG uptake similar to the insulin clamping technique, as previously reported by Knuuti et al. study.^14^ Accordingly, the FDG images showed a homogeneous distribution in all patients. The PET protocol composed of a 20 minutes dynamic NH_3_ scan followed by a 40 minutes dynamic FDG scan at rest in list-mode acquisition, triggered with 16 gates, and during free breathing. Both NH3 and FDG were administered intravenously as a bolus.

The simultaneously acquired CMR protocol included steady state free precession cine sequences in 2, 3, and 4 chamber view, short axis view, left ventricular outflow tract (LVOT) for evaluation of ventricular function as well as late gadolinium enhancement using phase-sensitive inversion recovery (PSIR) sequences 10 minutes after injection of .15 mL/kg of gadolinium (Gadovist®), as all previously described in detail.^15^,^16^ All PET/CMR scans were performed at rest only.

### First-Pass Ejection Fraction of Right and Left Ventricle from FDG and NH_3_

To determine RV and LV first-pass ejection fraction (FPEF) from FDG and NH_3_ dynamic PET images, the first 2 minutes of each PET list-mode dataset were reconstructed by dividing them into 15 frames to visualize the time of first-pass of the tracer in the ventricles and creating count-rate plots over the entire heart. From this information, the proper start points and time duration of the first-pass in both ventricles was defined. Gated reconstruction of the first-pass was performed with vendor-based software (e7tools; Siemens Healthineers, Knoxville, USA) using ECG information for dividing the heart cycle into 8 gates. As reconstruction algorithm an ordinary-Poisson ordered-subsets-expectation-maximization (OP-OSEM) with point spread function resolution modeling, three iterations and 24 subsets was used. Data were corrected for attenuation, scatter, normalization, in frame decay, deadtime, and random. The reconstruction included a 4 mm FWHM Gaussian smoothing of the final images. In addition, a further 19 mm FWHM Gaussian smoothing was applied after reconstruction to achieve a smooth representation of the activity distribution within the ventricles.

Volumes-of-interest were placed around the RV and LV in the EDV, in general overestimating the true volume of the respective ventricle but trying to match with the cardiac valves. This was done by manual delineation slice-by-slice in the EDV frame by clinically experienced cardiac nuclear medicine specialists using the software Hermes Hybrid Viewer PDR, version 4.0.0 (HERMES Medical Solutions, Stockholm, Sweden) and merging the individual ROIs to the final VOIs for the RV and LV.

Under the assumption that the activity in the myocardium and the background can be neglected compared to the activity in the ventricles during the FP, the total activity within these VOIs corresponds to the total activity within the respective ventricles. Therefore, the total activity in the VOIs were exported in the EDV and ESV gate to obtain the total activity (A_total_) in the respective ventricle in both, the ED and ES phase. Under the assumption that the activity concentration (*A*_cons_) of the blood does not changed during one heart cycle the EF could then be calculated following:

$$ {\text{EF}} = \frac{{ {\text{EDV}} - {\text{ESV}}}}{{   {\text{EDV}}}} \times 100 = \frac{{ {\text{EDV}} - {\text{ESV}}}}{{   {\text{EDV}}}} \times \frac{{ A_{\text{cons}} }}{{  A_{\text{cons}} }} \times 100 = \frac{{ A_{{{\text{total }}\,{\text{EDV}}}} -  A_{{{\text{total }}\,{\text{ESV}}}}  }}{{ A_{{{\text{total}}\,{\text{EDV}}}}  }} \times 100 $$

**Figure 1:**
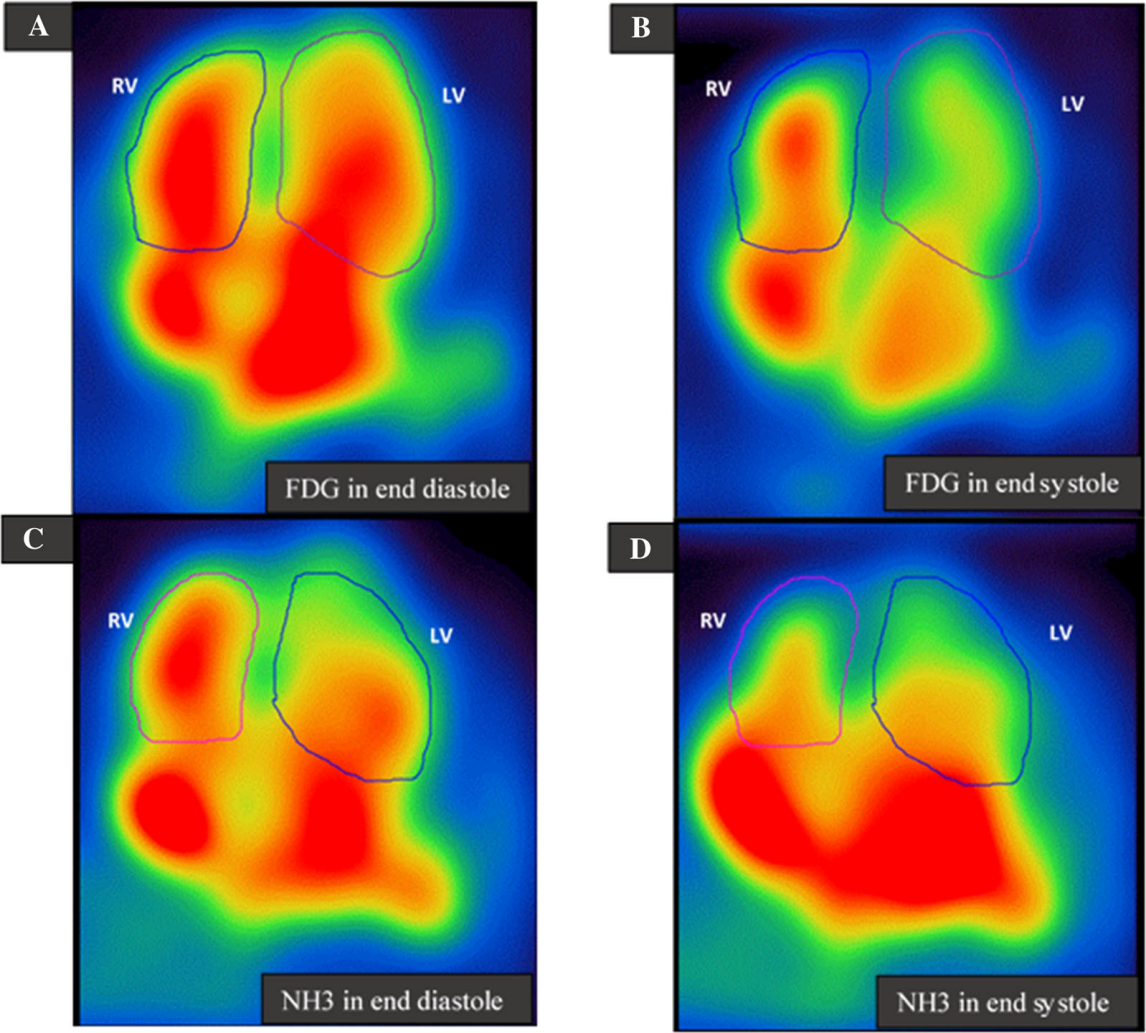
Manual delineation of the right (RV) and left ventricle (LV) from the EDV frames for estimation of first-pass ejection fraction of the left and right ventricle in a 74-year patient with ischemic coronary heart disease. (A) FDG in end-diastolic (ED) and (B) in end-systolic (ES) phase. (C) NH_3_ in ED and (D) in ES

The entire manual segmentation procedure was applied for each patient and for both FDG (Figure 1A, B) and NH_3_ (Figure 1C, D).

### Early- and Late-Phase Left Ventricular Function and Volumes

**Figure 2:**
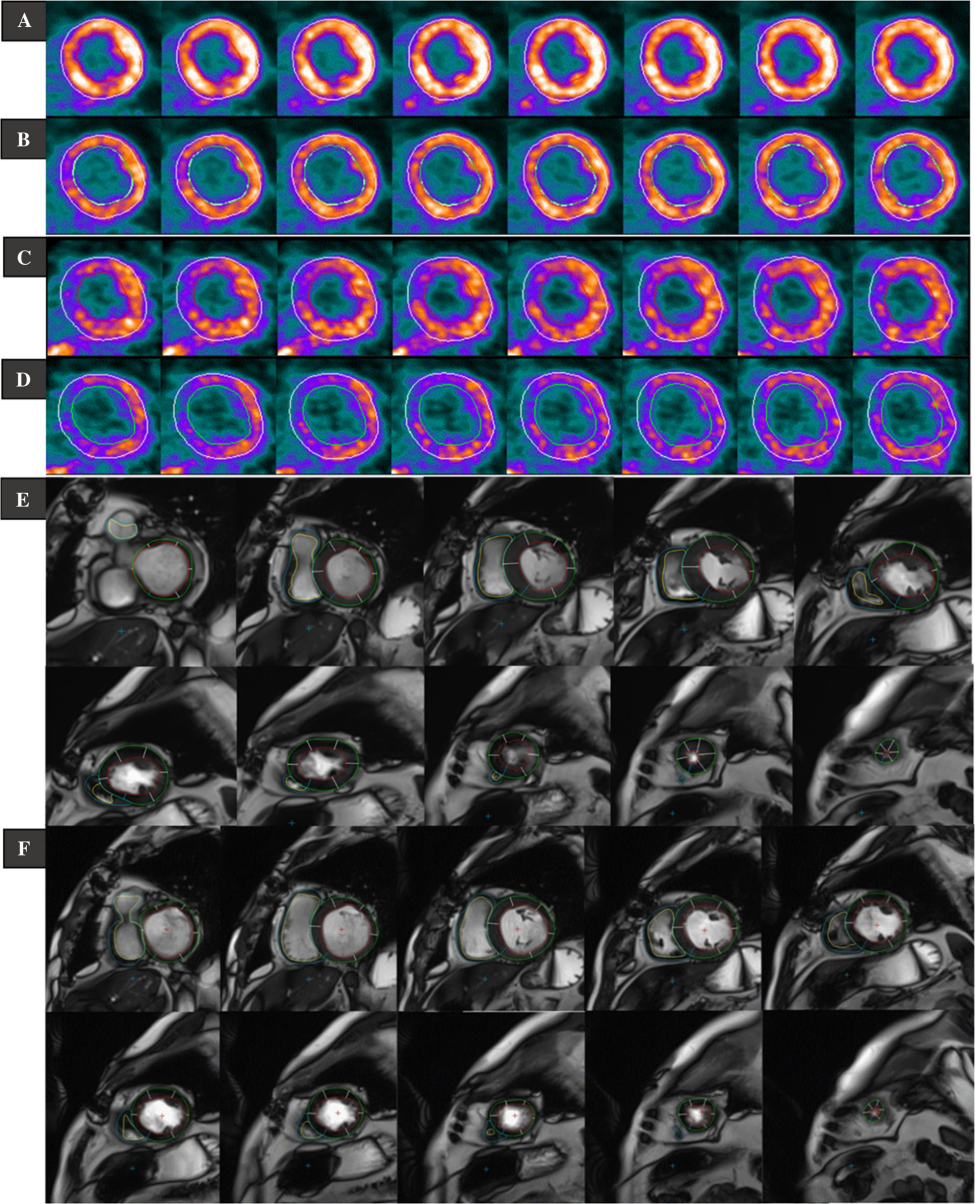
Automated delineation of the left ventricle (LV) for estimation of late-phase cardiac function of left using the software Cedars-Sinai Medical Center: (A) FDG in end systole and (B) in end diastole. (C) NH_3_ in end systole and (D) in end diastole. Manual delineation of left and right ventricle in CMR of both right ventricle (RV) and LV: (E) in end systole and (F) in end diastole

Quantitative analysis of both FDG and NH_3_ PET data for obtaining the left ventricular parameters such as end-systolic volume (LVESV), end-diastolic volume (LVEDV), and ejection fraction (LVEF) from early- (EP) and late-phase (LP), i.e., myocardial-uptake, images was conducted using the software QGS® (Version 2013.3, Cedars-Sinai Medical Center, Los Angeles, USA). Applying count-based calculations and automated delineation of myocardium, the software has measured the myocardial EF from ESV and EDV for the left ventricle automatically of FDG PET (Figure 2A, B) and of NH_3_ PET (Figure 2C, D). For this reason, dynamic ECG-gated images from EP (first 2 to 10 minutes) in 8 and 16 gates and LP (last 10 minutes) of both PET examinations were reconstructed, reoriented, and post-filtered.

### CMR Left and Right Ventricular Function and Volumes

The quantitative analysis of CMR from DICOM images for acquiring LVESV, LVEDV, LVEF and RVEF was achieved by an experienced CMR radiologist according to the MR guidelines of cardiovascular MRI image interpretations.^17^ All CMR parameters were then post processed using a dedicated cardiac postprocessing software (QMass®, MEDIS, Leiden, Netherlands). RV- and LVEDV were defined as to have the largest RV and LV blood volume, respectively. The RV- and LVESV were outlined with the smallest RV and LV blood volume, respectively (Fig 2E, F).

### Statistical Analysis

The IBM SPSS Software version 24.0 was used for all data entry and analysis. All obtained data were tested for normality of distribution using the Kolmogorov–Smirnov test. Parameters that showed a normal distribution were presented in mean ± standard deviation (SD). Not normally distributed parameters, if available, were presented in median ± (minimal - maximal) range and were log_10_-transformed for analysis. Categorical variables were shown in percentages and number of recorded cases, as stated. Pearson’s correlations coefficient was used to study correlations of PET (FDG and NH_3_) parameters with CMR parameters, which were considered as gold standard. Hence, a correlation coefficient (*R*) of ≥ .5 and < .6 was considered weak, a *R* ≥ .6 and < .8 was considered moderate and a *R* ≥ .8 was considered strong.^18^ Furthermore, we performed a Bland-Altman plot analysis between PET and CMR concerning FP parameters to additionally show the agreement between the two devices. For all statistical analysis, a *P*-value of < .05 was considered statistically significant.

## Results

Twenty-two consecutive patients (77% male, aged 69 ± 9 years, body mass index 27.3 ± 4.6 kg/m^2^), were eligible for evaluation and the clinical characteristics of these patients and their cardiovascular risk factors are shown in Table 1. Fourteen of 22 patients (64%) presented with CHD and 6 (36%) suffered from ischemic cardiomyopathy. Mean ± SD of blood glucose prior the FDG injection was 124 ± 33 mg/dL and the injected FDG and NH_3_ activities were 334 ± 35 and 816 ± 95 MBq, respectively. The mean of the heart rate of these patients was 68 ± 12 beats/minute. Seven patients (32%) had type 2 diabetes mellitus, merely 1 (14%) was insulin-dependent and 6 (86%) were treated with only oral antidiabetic therapies, mostly metformin. 77% of the evaluated patients presented with arterial hypertension, 64% with hyperlipidemia, and 14% were active smokers. Approximately 46% of the patients presented with at least one coronary stent and 32% with a coronary bypass graft. 23% of patients had prior myocardial infarction.

**Table 1:** Clinical characteristics of study participants (N = 22)

Parameters	Values
Patients (N)	22
Male (N) (%)	(17) 77%
Female (N) (%)	(5) 23%
Age (mean ± SD) year	69 ± 9
Underlying cardiac diseases	
CHD (N) (%)	(14) 64
ICMP (N) (%)	(8) 36
Height, mean ± SD (cm)	175 ± 10
Weight, mean ± SD (kg)	84 ± 17
Body mass index, mean ± SD (kg/m^2^)	27.3 ± 4.6
NH_3_, mean ± SD (MBq)	816 ± 95
FDG, mean ± SD (MBq)	334 ± 35
Blood glucose pre FDG injection, mean ± SD (mg/dl)	124 ± 33
Heart rate beats/minutes	68 ± 12
Lipid parameters (mean ± SD)	
Triglyceride	162 ± 68
Total cholesterol	149.5 ± 55
LDL	43 ± 12.9
HDL*	81.6 (46–132)
Type 2 diabetes mellitus (N) (%)	(7) 32
Insulin-dependent (N) (%)	(1) 14
On OADs (N) (%)	(6) 86
Cardiovascular risk factors	
Arterial hypertension (N) (%)	(17) 77.3
Hyperlipidemia (N) (%)	(14) 63.6
Smoking status (N) (%)	
Current	(3) 13.6
Former	(6) 27.3
Never	(7) 31.8
Unknown	(6) 27.7
Previous history (N) (%)	
Coronary stents	(10) 45.5
Bypass operation	(7) 31.8
Myocardial infarction	(5) 22.7

*HDL* high density lipoprotein; *ICMP* ischemic cardiomyopathy; *LDL* low density lipoprotein; *OADs* oral antidiabetics

*Not normally distributed and presented in median ± (minimal − maximal) range

### Functional Parameters Acquired from FP FDG and NH_3_ PET and from CMR

The RV and LV FPEF obtained from FDG and NH_3_ were 28% ± 11 and 32% ± 15 as well as 32% ± 11 and 41% ± 14, respectively. The RVEF and the LVEF received from the CMR were 42% ± 16 and 45% ± 19, respectively (Table 2).

**Table 2:** Ejection fraction of the right and left ventricle assessed with dynamic gated FDG and NH_3_ PET both in first 2 minutes of image acquisition and with CMR in an integrated PET/MRI (N = 22)

Image modality	First-pass (0–2 minutes)	
	Right ventricle	Left ventricle
Gated FDG		
FPEF % (mean ± SD)	28 ± 11^#^	32 ± 15^#^
Gated NH_3_		
FPEF % (mean ± SD)	32 ± 11^§^	41 ± 14
CMR		
EF % (median + range)*	43 (9–62)^#,§^	45 ± 19^#^

*FPEF*, first-pass ejection fraction; *SD*, standard deviation

*Not normally distributed and presented in median + range (minimal − maximal)

^#^Significant differences between CMR and FDG parameters (*P* < .001)

^§^Significant differences between CMR and NH_3_ parameters (*P* < .001)

### Functional Parameters Acquired from Early- and Late-Phase FDG and NH_3_ PET and from CMR

As presented in Table 3, the LVESV as well as LVEDV and LVEF acquired from EP dynamic FDG in 8 and 16 gates were [71 (15 to 213 mL), 98 (16 to 241 mL) and 32 ± 17%] and [50 (17 to 206 mL), 93 (13 to 219 mL), and 36 ± 17%], respectively, while LVESV, LVEDV and LVEF acquired from LP dynamic FDG were 85 ± 63 mL, 138 ± 63 mL, and 47 ± 19%, respectively.

**Table 3:** Parameters of left ventricular function assessed with QGS software obtained from early (8 and 16 gated) and late imaging phase of FDG and NH_3_ PET scan and from CMR in an integrated PET/MRI (N = 22)

Image modality	Parameters of the left ventricular function		
	ESV (mL)	EDV (mL)	EF (%)
FDG (mean ± SD)			
Early-phase (2–10) minutes			
8 gates	71 (15–213)	98 (16–241)*^,#^	32 ± 17^#^
16 gates	50 (17–206)*^,#^	93 (13–219)*^,#^	36 ± 17^#^
Late-phase (last 10) minutes	85 ± 63	^#^138 ± 63	47 ± 19
NH_3_ (mean ± SD)			
Early-phase (2–10) minutes			
8 gates	60 (19–360)	109 (56–384)*^,§^	41 ± 22
16 gates	54 (16–371)*	116 (57–431)*^,§^	46 ± 24
Late-phase (last 10) minutes	79 ± 56	137 ± 63	47 ± 20
CMR (mean ± SD)	93 ± 66^#,§^	153 ± 71^#,§^	45 ± 19^#^

*ESV*, end-systolic volume; *EDV*, end-diastolic volume; *EF*, ejection fraction; *SD*, standard deviation

*Not normally distributed and presented in median + range (minimal − maximal)

^#^Significant differences between CMR and FDG parameters (*P* < .01)

^§^Significant differences between CMR and NH_3_ parameters (*P* < .001)

Moreover, the LVESV, LVEDV and LVEF measured from EP dynamic NH_3_ in 8 and 16 gates were [60 (19 to 360 mL), 109 (56 to 384 mL), and 41 ± 22%] and [54 (16 to 371 mL), 116 (57 to 431 mL), and 46 ± 24%], respectively and from LP dynamic NH_3_ were 79 ± 56 mL, 137 ± 63 mL, and 47 ± 20%, respectively (Table 3).

The LVESV, LVEDV and LVEF acquired from CMR during the integrated PET/MRI examination were 93 ± 66 mL, 153 ± 71 mL and 45 ± 19%, respectively, all in Table 3.

### LV and RV FPEF acquired from FDG and NH_3_ PET compared to CMR

**Figure 3:**
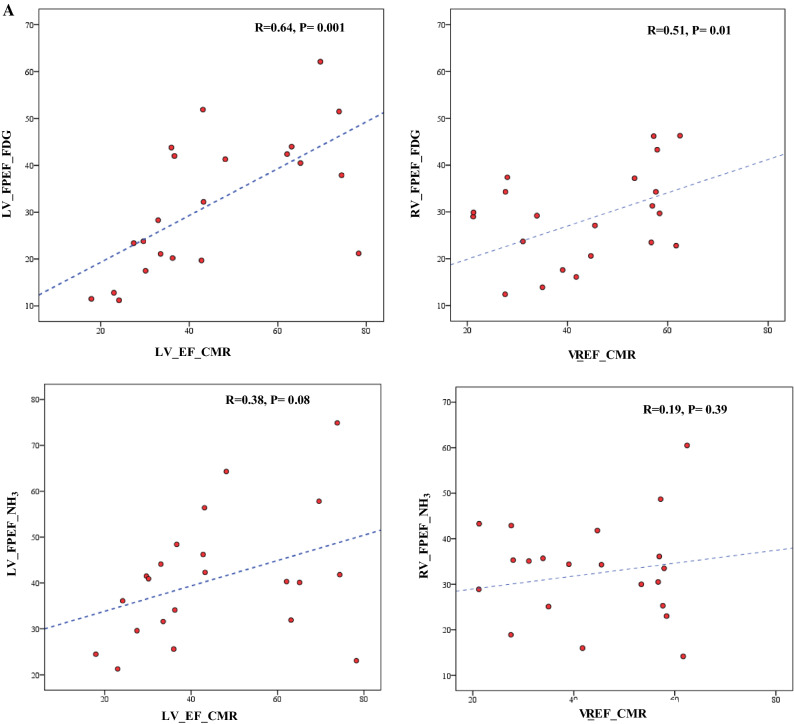

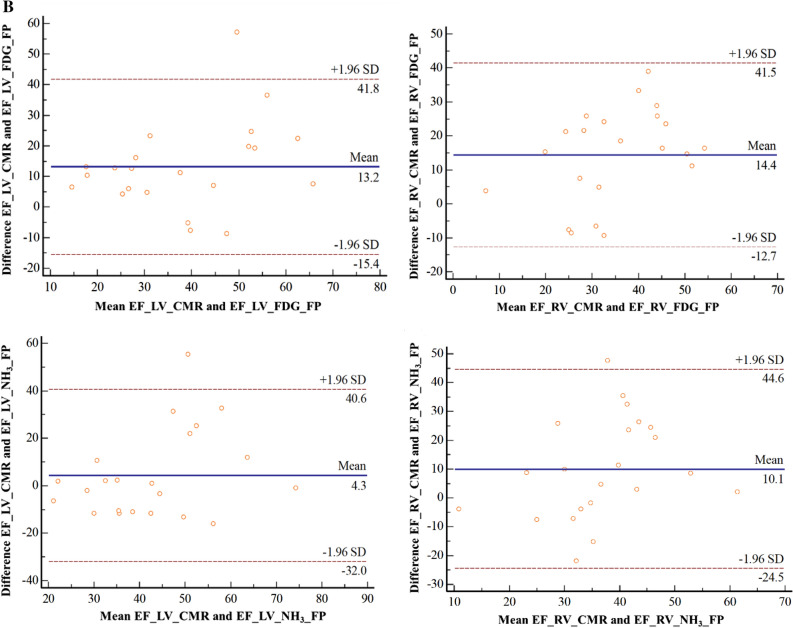
(**A**) Intra-individual correlation of LV and RV FPEF acquired from FDG and NH_3_ PET with CMR in an integrated PET/MRI system. LV: left ventricle; RV: right ventricle FPEF: first-pass ejection fraction; CMR: cardiac magnetic resonance imaging. LV and the RV FPEF obtained from FDG PET showed positively moderate (*R* = .64, *P* = .01) and weak (*R* = .51, *P* = .01) correlations with that received from CMR. No significant correlations were observed between LV and RV FPEF from NH_3_ PET and CMR. (**B**) Intra-individual agreements with Bland Altmann analysis of LV and RV FPEF acquired from FDG and NH_3_ PET with CMR in an integrated PET/MRI system. *LV*, left ventricle; *RV*, right ventricle FPEF: first-pass ejection fraction; *CMR*, cardiac magnetic resonance imaging. Bland Altmann analysis shows a higher bias of about 14% EF (EF unit) for CMR compared to FDG PET and about 10% higher EF compared to NH_3_ PET

Although results revealed significant differences between EF of CMR and FPEF of both FDG and NH3 (*P* < .001), the LV and the RV FPEF obtained from FDG PET showed positively moderate and weak correlations with the LVEF and RVEF received from CMR (*R* = .64, *P* = .01) and (*R* = .51, *P* = .01) respectively. Nevertheless, no significant correlations were observed between LV and RV FPEF obtained from NH3 PET scan as compared to LVEF and RVEF from CMR, all Fig 3A. The Bland Altmann analysis for the agreement between the PET and CMR concerning these parameters showed a higher bias of about 14% EF (EF unit) for CMR compared to FDG PET and of about only 10% higher EF compared to NH_3_ PET (Fig 3B).

### LV Parameters from 8 and 16 Gates of EP Dynamic FDG and NH_3_ Compared to CMR

**Figure 4:**
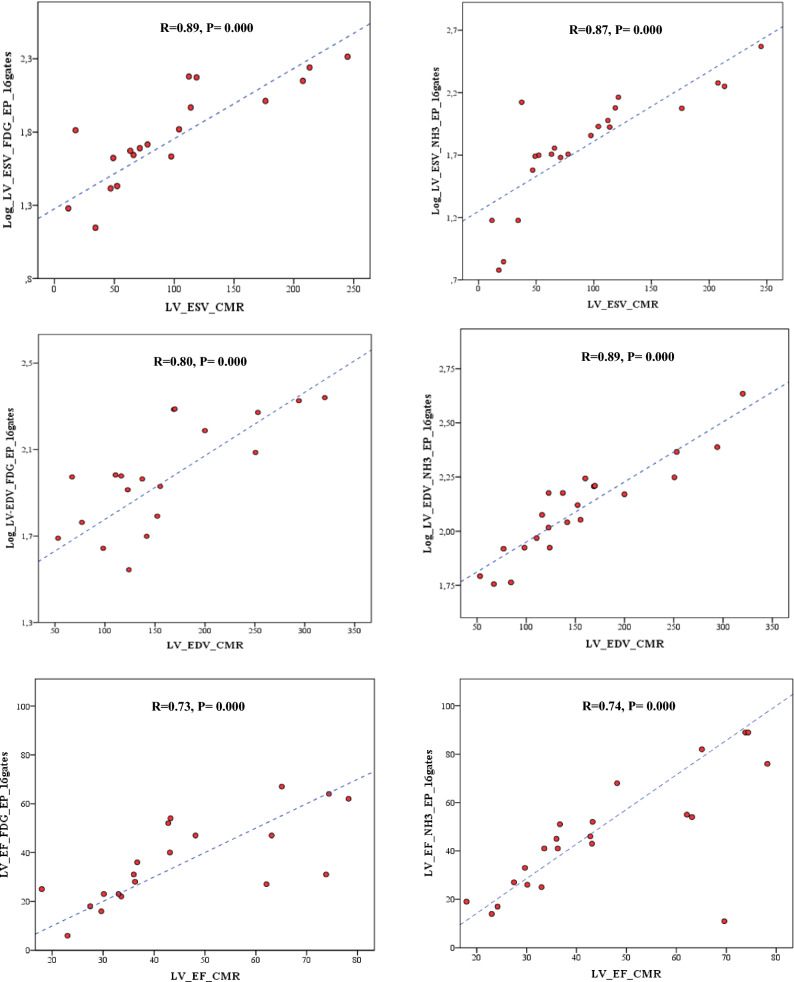
Intra-individual correlations of LVESV, LVEDV, and LVEF acquired from EP of dynamic FDG and NH3 PET in 16 gates as compared to CMR in an integrated PET/MRI system. *LV*, left ventricle; *RV*, right ventricle; *ESV*, end-systolic volume; *EP*, early-phase; *EDV*, end-diastolic volume; *EF*, ejection fraction; *CMR*, cardiac magnetic resonance imaging. All LV parameters moderately to strongly correlated to CMR when they acquired from EP FDG and NH_3_ in 16 gates (all *R* > .7 and *P* = .000)

We observed significant differences between CMR and all LV parameters measured from early 2 to 10 minutes of FDG PET in 8 and 16 gates (all *P* < .01). Furthermore, there were significant differences between CMR and LV parameters from early 2 to 10 minutes NH_3_ PET in 8 and 16 gates (*P* < .001), except for ESV in 16 gates and EF values in both 8 and 16 gates. However, all the LV parameters moderately to strongly correlated to CMR when they acquired from EP FDG and NH_3_ in 16 gates (all *R* > .7 and *P* = .000), Figure 4.

### LV Parameters from LP Dynamic FDG and NH_3_ PET Compared to CMR

**Figure 5:**
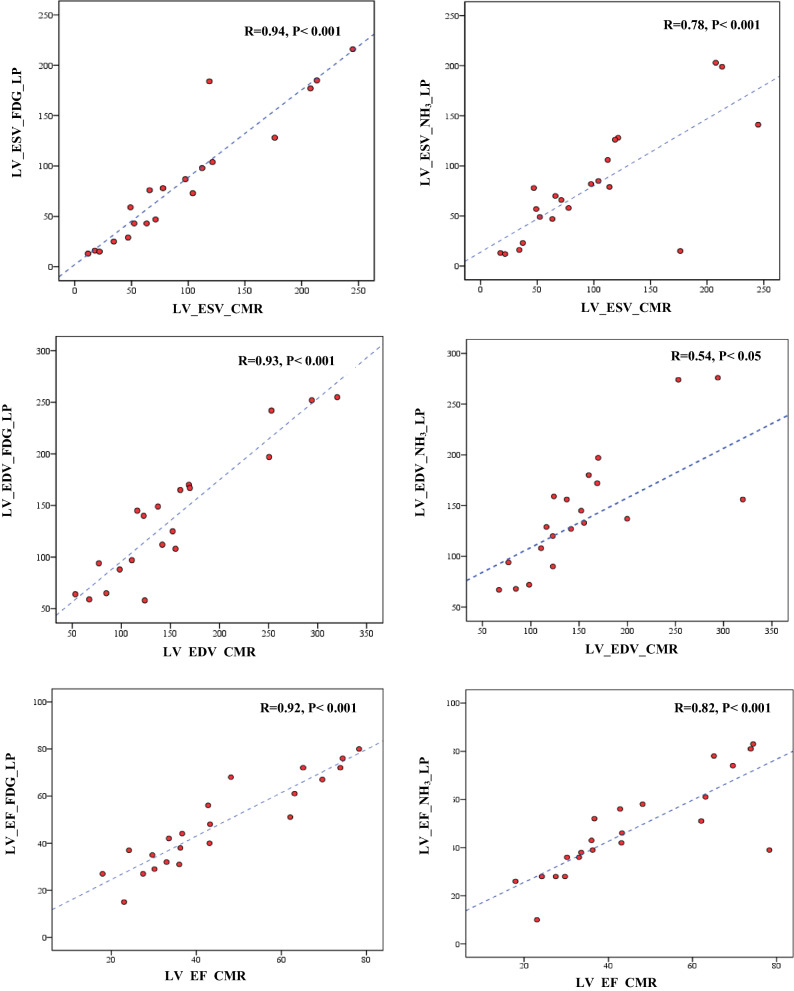
Intra-individual correlations of LVESV, LVEDV, and LVEF acquired from LP of dynamic FDG and NH_3_ PET as compared to CMR in an integrated PET/MRI system. *LV*, left ventricle; *RV*, right ventricle; *ESV*, end-systolic volume; *LP*, late-phase; *EDV*, end-diastolic volume; *EF*, ejection fraction; *CMR*, cardiac magnetic resonance imaging. All LV parameters acquired from LP FDG and NH3 correlated good to strongly positive with those received from CMR (all *R* > .7, and *P* < .001), except EDV from LP NH_3_ weakly correlated to the EDV of CMR (*R* = .54, *P* < .05)

There were no significant differences between LV parameters from CMR and LP dynamic from both FDG and NH_3_ PET, except for EDV obtained from FDG. In total, all LV parameters that acquired from LP dynamic FDG and NH_3_ correlated good to strongly positive with those received from CMR (all *R* > .7, and *P* < .001), except EDV from LP NH_3_ weakly correlated to the EDV of CMR (*R* = .54, *P* < .05), all shown in Figure 5.

## Discussion

In a cohort of patients with advanced CHD, we used an integrated PET/MRI system to intra-individually compare cardiac functional parameters obtained from dynamic ECG-gated FDG and NH_3_ PET scans with 3-Tesla-MRI functional parameters as a standard reference.

While the first-pass FDG data showed significant correlations between LVFPEF and RVFPEF with those gained from CMR, this was not the case for NH_3_. However, a systemic underestimation of the EF was found for both FDG (14%) and NH_3_ (10%). In this respect, Todica et al. previously acquired ECG-gated FDG PET examinations in seven healthy rats and found a good correlation between LV FPEF from FDG PET and LVEF values from CMR.^9^ In their study, functional parameters were calculated with the QBS^®^ software (Cedars-Sinai, Los Angeles, USA) for evaluating gated FDG blood-pool PET in the studied rats. Similarly, in 24 oncological patients, Bouallègue et al. could recently show possibility of assessing cardiac function from the gated FDG PET studies.^11^ They revealed a very good agreement between the results of LV FPEF obtained from gated FDG PET and those acquired from SPECT studies with equilibrium radionuclide angiography, where LV FPEF determined with an automatic segmentation software. Indeed, our current study is the first clinical research demonstrating the feasibility of calculating LV FPEF and RV FPEF from dynamic FDG and NH_3_ PET scans in patients with CHD. The disparities in correlations of FDG and NH_3_ PET with CMR observed in this study might be caused by the fact that both these tracers have different uptake-mechanisms in the myocardium. Due to the more rapid first-pass uptake of NH_3_ into the myocardium,^19^ thus, the assumption that the activity in the myocardium can be neglected during the first-pass to be able to calculate the EF form an extended VOI around the ventricle may not be entirely correct and particularly with NH_3_. Moreover, the different count statistics within the NH_3_ images may affected the manual ventricle delineation. Furthermore, the relatively high activity concentration of NH_3_ (816 ± 95 MBq) applied in our studied cohort should be taken into consideration, as this might increase the number of the detected random events and influencing the deadtime in the PET/MR detector system and affecting the reading of NH_3_ images.^20^ Nevertheless, the significant association of right and left ventricular FPEF from dynamic ECG-gated FDG examinations with that from CMR might enable concomitant assessment of cardiac function while performing a whole-body oncological FDG PET/MRI or even PET/CT. Basically, FDG PET scans using PET/CT or PET/MRT systems are frequently performed worldwide in a wide range of diagnostic areas,^21^ especially in oncology. Performing a dynamic blood-pool PET acquisition over the heart would allow the first-pass acquisition of cardiac functional parameters, which are important to early detect organ damage under the effect of cardiotoxic oncological therapies.

Moreover, results of the early- and late-phase analyses showed overall good to strong correlations between the left ventricular functional parameters such as LVESV, LVEDV, and LVEF acquired from both NH_3_ and FDG PET scans with that of CMR. In fact, because NH_3_ is a tracer commonly used to determine myocardial blood flow,^22^ studies that have directly compared cardiac functional parameters obtained from NH_3_ PET with those acquired from CMR are very limited. In a very recent 3T hybrid PET/MRI study, Nazir et al. could rather demonstrate a feasible quantification of myocardial blood flow with good to moderate agreement between PET and CMR in a phantom and five healthy volunteers undergoing adenosine stress, where NH_3_ and gadolinium were administered simultaneously.^23^ In an earlier study, Okazawa et al. showed in a subgroup of the examined participants with cardiovascular diseases a good agreement of the LVEF from NH_3_ PET with that obtained from left ventriculography.^24^ There are, albeit, some studies that found precise determination of cardiac function from ECG-gated NH_3_, when FDG cardiac function values were considered as gold standard.^25^,^26^

Furthermore, results from previous preclinical and human studies acquired with separate PET and CMR scanners using the late-phase FDG PET are in one line with the present study.^27^–^29^ Stegger et al. revealed in a preclinical study with 33 mice, mostly with occlusions of at least one coronary artery, a good agreement of cardiac function measures from FDG PET with that of CMR.^30^ Todica et al. were able to prove this agreement between FDG PET and CMR in healthy mice as well.^9^ Similar correlations were found for the comparison between [^68^Ga]Albumin PET, an experimental blood-pool marker, and CMR.^31^ Furthermore, excellent correlations for LV myocardial function were observed between FDG and CMR in a human study by Slart et al. that included 38 patients with chronic coronary heart diseases received separately gated FDG PET and CMR^7^ and in a study by Li et al. with patients with heart failure.^8^ Altogether, these results reveal that left ventricular parameters obtained from late-phase NH_3_ PET and right and left ventricular parameters acquired from first-pass and late-phase gated FDG PET examination can accurately be used to evaluate cardiac function.

Nonetheless, the retrospective design and the small sample size of the included cardiac PET/MRI scans, among them data of 32% type 2 diabetic patients, might limit the results of this study. Moreover, the used activity of about 800 MBq was above the peak noise equivalent count rate (NEC) of the system.^32^ Therefore, the absolute values of the activity concentrations measured during the first-pass are biased, as we cannot assume that all corrections (e.g., deadtime) work well at these high activity levels. However, we expect that this bias is a constant multiplicative factor for the 8 gates used for the calculation of the EF from the fist-pass images. Therefore, the bias cancels out in the formula (EDV × bias − ESV × bias)/EDV × bias = (EDV − ESV) × bias/EDV × bias = (EDV − ESV)/EDV and an eventual bias should not have any effect on the calculation of the EF. Additionally, results of this intra-individual comparison might have been influenced by the reader-dependent delineation of the ventricles of FP PET and particularly the RV, which is already considered a challenge,^33^ as an inaccurate ROI drawing might lead to inappropriate determination of the RV and LV contours in PET and MRI. Therefore, manual delineations using an automatic or semi-automatic delineating technique as that used in QBS® software might reduce reader dependence and further improve FP PET measures correlations with that of CMR. Finally, even it is unlikely that the blood-pool phase/early-phase of FDG distribution is significantly affected by different protocols and patient preparation, the transfer of our results into an oncological setting has to be made with caution.

## New Knowledge Gained

Based on the results of this study, deriving reliable LV and RV functional parameters from various examination phases of the dynamic NH_3_ and FDG is feasible and could potentially be estimated from other, mostly oncological routine scans when dynamic list-mode FDG PET acquisition is performed.

## Conclusions

Findings of this intra-individual PET/MRI analysis in patients with advanced CHD demonstrated the feasibility to calculate left and right ventricular function from several phases of dynamic NH_3_ and FDG PET scans using the first 2 as well as early 2 to 10 and late 10 minutes of PET list-mode data. There was a good agreement between the left ventricular ejection fraction calculated from the first-pass FDG PET acquisition and CMR, which could potentially open new perspectives for dynamic PET acquisitions in the oncological field or novel total body PET applications.

## Electronic supplementary material

Below is the link to the electronic supplementary material.

Electronic supplementary material 1 (PPTX 1081 kb)

## Abbreviations

CHD: Coronary heart disease
CMR: Cardiac magnetic resonance imaging
ESV: End-systolic volume
EDV: End-diastolic volume
EP: Early-phase
FDG: [^18^F]FDG
FPEF: First-pass ejection fraction
LP: Late-phase
LV: Left ventricle
LVEF: Left ventricular ejection fraction
NH_3_: [^13^N]NH_3_
PET: Positron emission tomography
PET/MRI: Positron emission tomography/magnetic resonance imaging
ROI: Region of interest
RV: Right ventricle
VOI: Volume of interest

## References

[1] Roth GA, Johnson C, Abajobir A, Abd-Allah F, Abera SF, Abyu G (2017). Global, regional, and national burden of cardiovascular diseases for 10 causes, 1990 to 2015. J Am Coll Cardiol.

[2] Nensa F, Bamberg F, Rischpler C, Menezes L, Poeppel TD, la Fougere C (2018). Hybrid cardiac imaging using PET/MRI: a joint position statement by the European Society of Cardiovascular Radiology (ESCR) and the European Association of Nuclear Medicine (EANM). Eur Radiol.

[3] Ghosh N, Rimoldi OE, Beanlands RS, Camici PG (2010). Assessment of myocardial ischaemia and viability: role of positron emission tomography. Eur Heart J.

[4] Kostakoglu L, Agress H, Jr., Goldsmith SJ. Clinical role of FDG PET in evaluation of cancer patients. Radiographics. 2003;23:315-40; quiz 533. 10.1148/rg.232025705 12640150

[5] Broder H, Gottlieb RA, Lepor NE (2008). Chemotherapy and cardiotoxicity. Rev Cardiovasc Med.

[6] Fraum TJ, Fowler KJ, McConathy J (2016). PET/MRI: Emerging clinical applications in oncology. Acad Radiol.

[7] Slart RH, Bax JJ, de Jong RM, de Boer J, Lamb HJ, Mook PH (2004). Comparison of gated PET with MRI for evaluation of left ventricular function in patients with coronary artery disease. J Nucl Med.

[8] Li Y, Wang L, Zhao SH, He ZX, Wang DY, Guo F (2014). Gated F-18 FDG PET for assessment of left ventricular volumes and ejection fraction using QGS and 4D-MSPECT in patients with heart failure: A comparison with cardiac MRI. PLoS ONE.

[9] Todica A, Boning G, Lehner S, Weidl E, Cumming P, Wangler C (2013). Positron emission tomography in the assessment of left ventricular function in healthy rats: a comparison of four imaging methods. J Nucl Cardiol..

[10] Ben Bouallegue F, Mariano-Goulart D, Agostini D, Manrique A (2018). Feasibility of biventricular volume and function assessment using first-pass gated (15)O-water PET. EJNMMI Res.

[11] Ben Bouallegue F, Maimoun L, Kucharczak F, Le Fur P, Vauchot F, Hay B (2019). Left ventricle function assessment using gated first-pass (18)F-FDG PET: Validation against equilibrium radionuclide angiography. J Nucl Cardiol..

[12] Dilsizian V, Bacharach SL, Beanlands RS, Bergmann SR, Delbeke D, Dorbala S (2016). ASNC imaging guidelines/SNMMI procedure standard for positron emission tomography (PET) nuclear cardiology procedures. J Nucl Cardiol.

[13] Chang SA, Kim RJ (2016). The use of cardiac magnetic resonance in patients with suspected coronary artery disease: A clinical practice perspective. J Cardiovasc Ultrasound.

[14] Knuuti MJ, Yki-Jarvinen H, Voipio-Pulkki LM, Maki M, Ruotsalainen U, Harkonen R (1994). Enhancement of myocardial [fluorine-18]fluorodeoxyglucose uptake by a nicotinic acid derivative. J Nucl Med.

[15] Beitzke D, Rasul S, Lassen ML, Pichler V, Senn D, Stelzmuller ME (2019). Assessment of myocardial viability in ischemic heart disease by PET/MRI: Comparison of left ventricular perfusion, hibernation, and scar burden. Acad Radiol.

[16] Lassen ML, Beyer T, Berger A, Beitzke D, Rasul S, Buther F (2019). Data-driven, projection-based respiratory motion compensation of PET data for cardiac PET/CT and PET/MR imaging. J Nucl Cardiol.

[17] Schulz-Menger J, Bluemke DA, Bremerich J, Flamm SD, Fogel MA, Friedrich MG (2013). Standardized image interpretation and post processing in cardiovascular magnetic resonance: Society for Cardiovascular Magnetic Resonance (SCMR) board of trustees task force on standardized post processing. J Cardiovasc Magn Reson.

[18] Akoglu H (2018). User’s guide to correlation coefficients. Turk J Emerg Med.

[19] Machac J (2007). Radiopharmaceuticals for clinical cardiac PET. Imaging..

[20] O’Doherty J, Chalampalakis Z, Schleyer P, Nazir MS, Chiribiri A, Marsden PK (2017). The effect of high count rates on cardiac perfusion quantification in a simultaneous PET-MR system using a cardiac perfusion phantom. EJNMMI Phys.

[21] Nensa F, Beiderwellen K, Heusch P, Wetter A (2014). Clinical applications of PET/MRI: current status and future perspectives. Diagn Interv Radiol.

[22] Kuhle WG, Porenta G, Huang SC, Buxton D, Gambhir SS, Hansen H (1992). Quantification of regional myocardial blood flow using 13N-ammonia and reoriented dynamic positron emission tomographic imaging. Circulation.

[23] Nazir MS, Gould SM, Milidonis X, Reyes E, Ismail TF, Neji R (2019). Simultaneous (13)N-ammonia and gadolinium first-pass myocardial perfusion with quantitative hybrid PET-MR imaging: A phantom and clinical feasibility study. Eur J Hybrid Imaging.

[24] Okazawa H, Takahashi M, Hata T, Sugimoto K, Kishibe Y, Tsuji T (2002). Quantitative evaluation of myocardial blood flow and ejection fraction with a single dose of (13)NH(3) and gated PET. J Nucl Med.

[25] Szymanski MK, Kruizinga S, Tio RA, Willemsen AT, Schafers MA, Stegger L (2012). Use of gated 13N-NH3 micro-PET to examine left ventricular function in rats. Nucl Med Biol.

[26] Khorsand A, Graf S, Eidherr H, Wadsak W, Kletter K, Sochor H (2005). Gated cardiac 13N-NH3 PET for assessment of left ventricular volumes, mass, and ejection fraction: comparison with electrocardiography-gated 18F-FDG PET. J Nucl Med.

[27] Schaefer WM, Lipke CS, Nowak B, Kaiser HJ, Reinartz P, Buecker A (2004). Validation of QGS and 4D-MSPECT for quantification of left ventricular volumes and ejection fraction from gated 18F-FDG PET: comparison with cardiac MRI. J Nucl Med.

[28] Khorsand A, Graf S, Frank H, Kletter K, Sochor H, Maurer G (2003). Model-based analysis of electrocardiography-gated cardiac (18)F-FDG PET images to assess left ventricular geometry and contractile function. J Nucl Med.

[29] Higuchi T, Nekolla SG, Jankaukas A, Weber AW, Huisman MC, Reder S (2007). Characterization of normal and infarcted rat myocardium using a combination of small-animal PET and clinical MRI. J Nucl Med.

[30] Stegger L, Heijman E, Schafers KP, Nicolay K, Schafers MA, Strijkers GJ (2009). Quantification of left ventricular volumes and ejection fraction in mice using PET, compared with MRI. J Nucl Med.

[31] Todica A, Brunner S, Boning G, Lehner S, Nekolla SG, Wildgruber M (2013). [68Ga]-albumin-PET in the monitoring of left ventricular function in murine models of ischemic and dilated cardiomyopathy: Comparison with cardiac MRI. Mol Imaging Biol.

[32] Delso G, Furst S, Jakoby B, Ladebeck R, Ganter C, Nekolla SG (2011). Performance measurements of the Siemens mMR integrated whole-body PET/MR scanner. J Nucl Med.

[33] Tulevski II, Romkes H, Dodge-Khatami A, van der Wall EE, Groenink M, van Veldhuisen DJ (2002). Quantitative assessment of the pressure and volume overloaded right ventricle: imaging is a real challenge. Int J Cardiovasc Imaging.

