# Supplementary material for: Assessment of left and right ventricular functional parameters using dynamic dual-tracer [13N]NH3 and [18F]FDG PET/MRI
Source: J Nucl Cardiol. 2020 Oct 22;29(3):1003–17. doi: 10.1007/s12350-020-02391-y (PMC9163002; doi:10.1007/s12350-020-02391-y)
Supplement: Supplementary file 1 — Electronic supplementary material 1 (PPTX 1081 kb) [file 12350_2020_2391_MOESM1_ESM.pptx]

## Slide 1
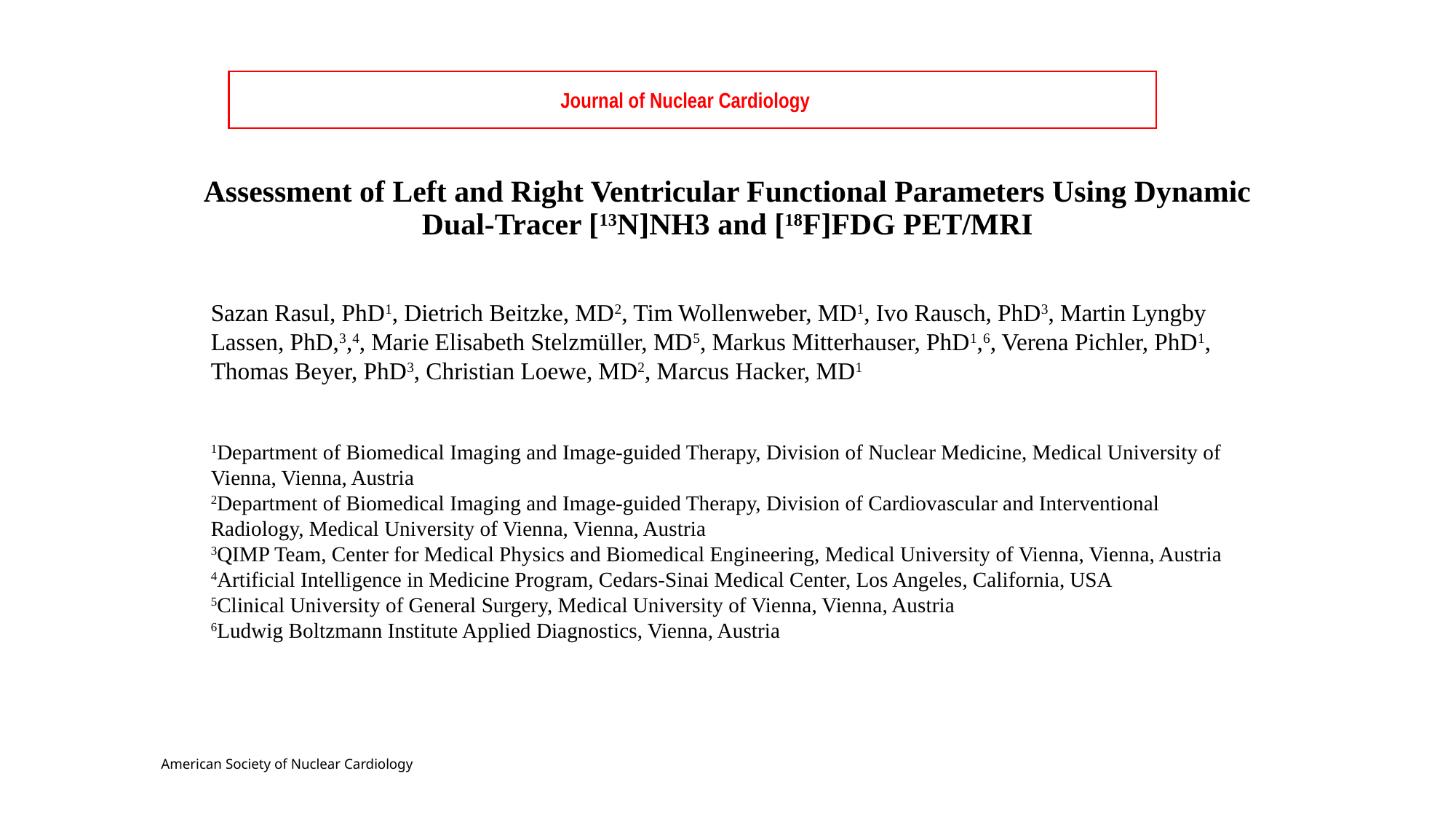

Journal of Nuclear Cardiology
Assessment of Left and Right Ventricular Functional Parameters Using Dynamic Dual-Tracer [13N]NH3 and [18F]FDG PET/MRI
Sazan Rasul, PhD1, Dietrich Beitzke, MD2, Tim Wollenweber, MD1, Ivo Rausch, PhD3, Martin Lyngby Lassen, PhD,3,4, Marie Elisabeth Stelzmüller, MD5, Markus Mitterhauser, PhD1,6, Verena Pichler, PhD1, Thomas Beyer, PhD3, Christian Loewe, MD2, Marcus Hacker, MD1
1Department of Biomedical Imaging and Image-guided Therapy, Division of Nuclear Medicine, Medical University of Vienna, Vienna, Austria
2Department of Biomedical Imaging and Image-guided Therapy, Division of Cardiovascular and Interventional Radiology, Medical University of Vienna, Vienna, Austria
3QIMP Team, Center for Medical Physics and Biomedical Engineering, Medical University of Vienna, Vienna, Austria
4Artificial Intelligence in Medicine Program, Cedars-Sinai Medical Center, Los Angeles, California, USA
5Clinical University of General Surgery, Medical University of Vienna, Vienna, Austria
6Ludwig Boltzmann Institute Applied Diagnostics, Vienna, Austria
American Society of Nuclear Cardiology

## Slide 2
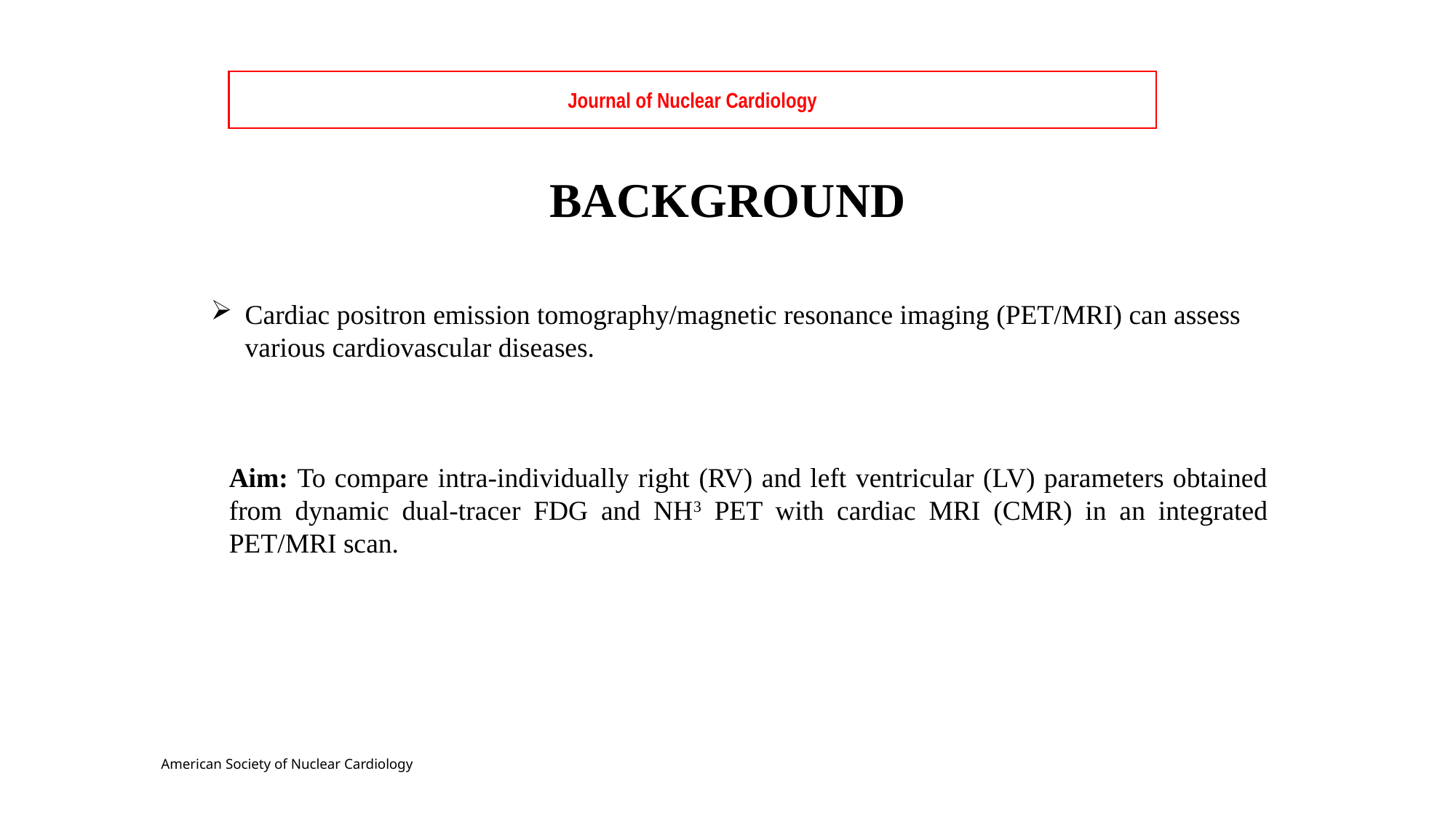

Journal of Nuclear Cardiology
BACKGROUND
Cardiac positron emission tomography/magnetic resonance imaging (PET/MRI) can assess various cardiovascular diseases.
Aim: To compare intra-individually right (RV) and left ventricular (LV) parameters obtained from dynamic dual-tracer FDG and NH3 PET with cardiac MRI (CMR) in an integrated PET/MRI scan.
American Society of Nuclear Cardiology

## Slide 3
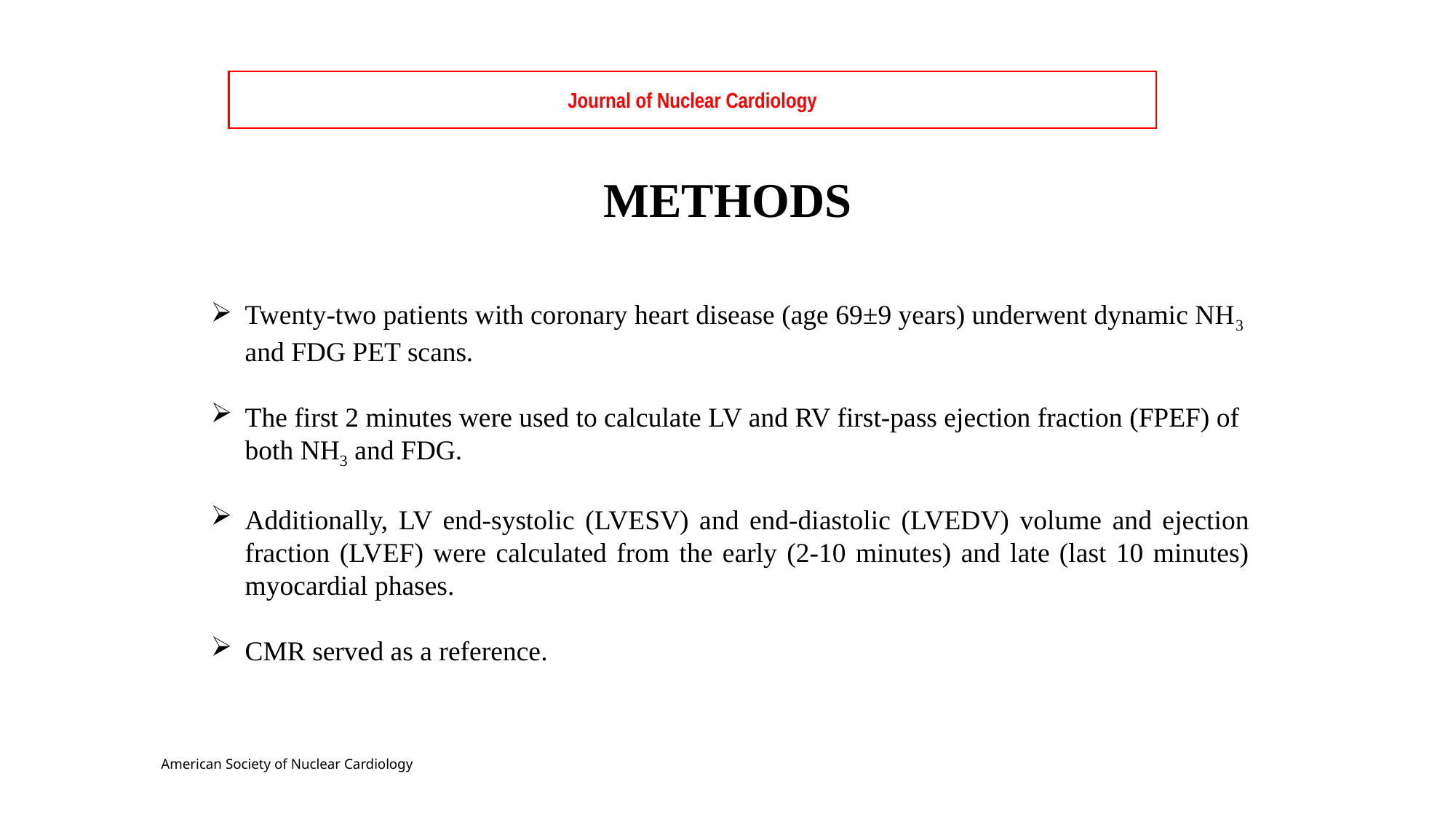

Journal of Nuclear Cardiology
METHODS
Twenty-two patients with coronary heart disease (age 69±9 years) underwent dynamic NH3 and FDG PET scans.
The first 2 minutes were used to calculate LV and RV first-pass ejection fraction (FPEF) of both NH3 and FDG.
Additionally, LV end-systolic (LVESV) and end-diastolic (LVEDV) volume and ejection fraction (LVEF) were calculated from the early (2-10 minutes) and late (last 10 minutes) myocardial phases.
CMR served as a reference.
American Society of Nuclear Cardiology

## Slide 4
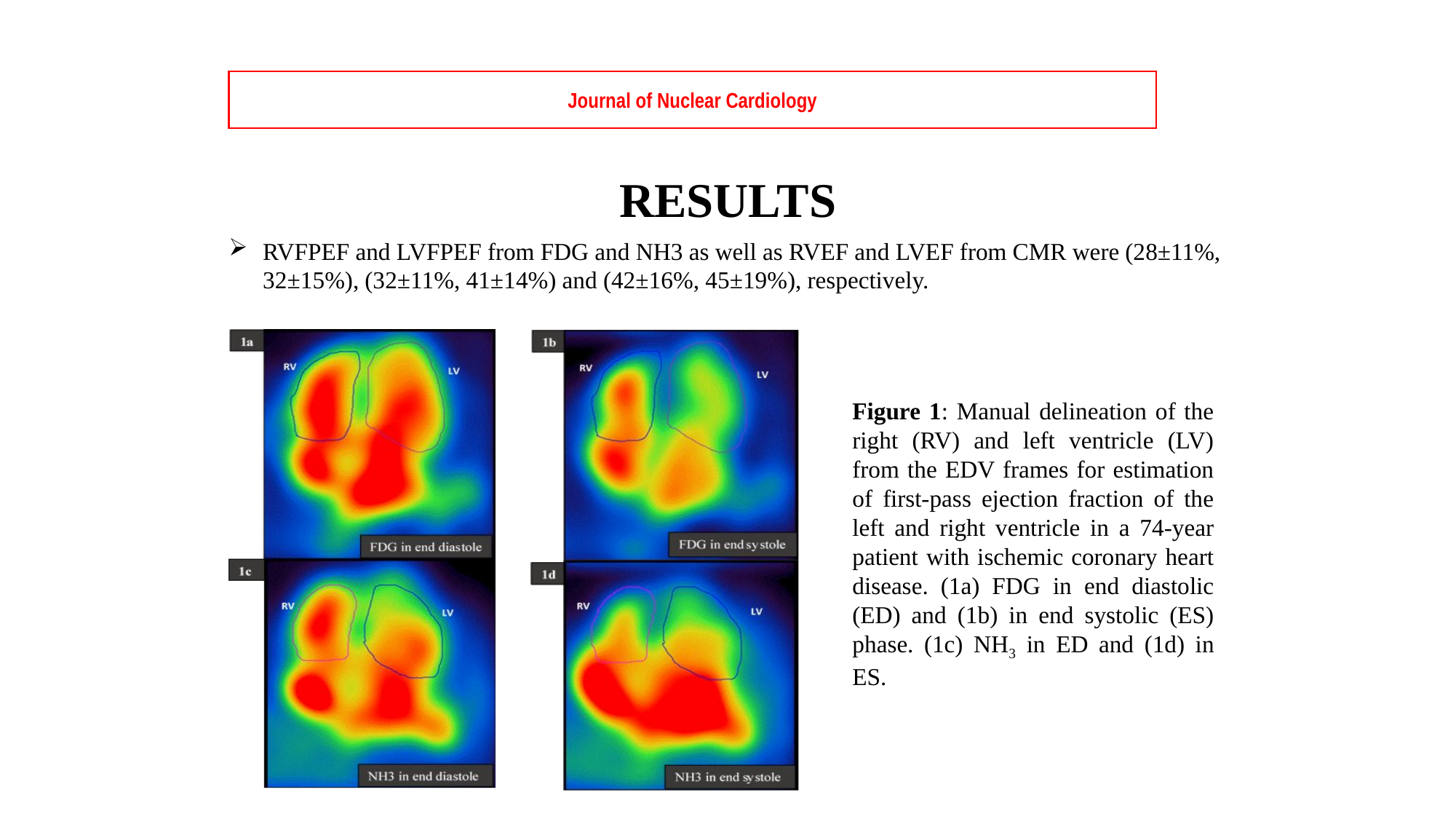

Journal of Nuclear Cardiology
RESULTS
RVFPEF and LVFPEF from FDG and NH3 as well as RVEF and LVEF from CMR were (28±11%, 32±15%), (32±11%, 41±14%) and (42±16%, 45±19%), respectively.
Figure 1: Manual delineation of the right (RV) and left ventricle (LV) from the EDV frames for estimation of first-pass ejection fraction of the left and right ventricle in a 74-year patient with ischemic coronary heart disease. (1a) FDG in end diastolic (ED) and (1b) in end systolic (ES) phase. (1c) NH3 in ED and (1d) in ES.

## Slide 5
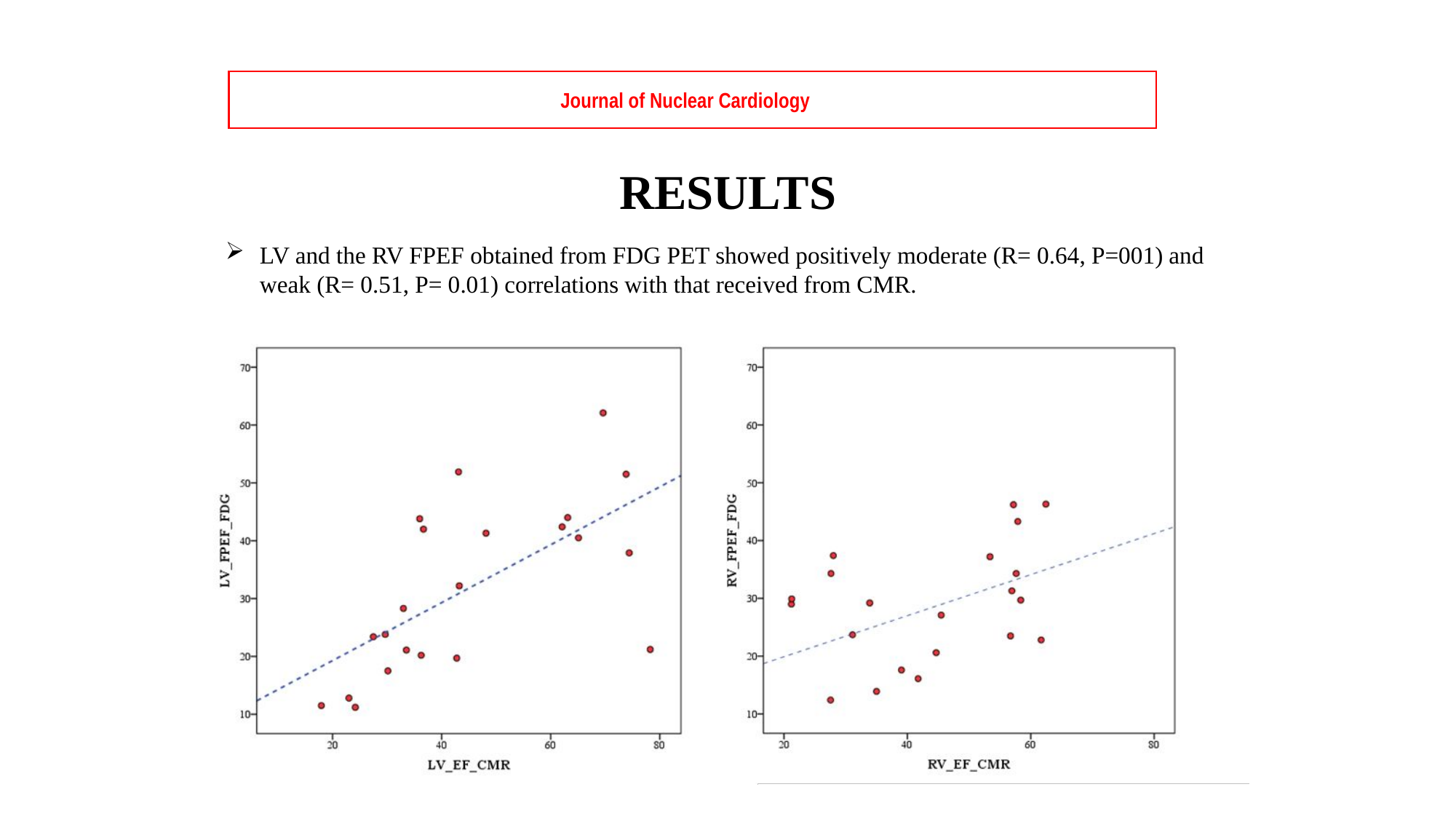

Journal of Nuclear Cardiology
RESULTS
LV and the RV FPEF obtained from FDG PET showed positively moderate (R= 0.64, P=001) and weak (R= 0.51, P= 0.01) correlations with that received from CMR.

## Slide 6
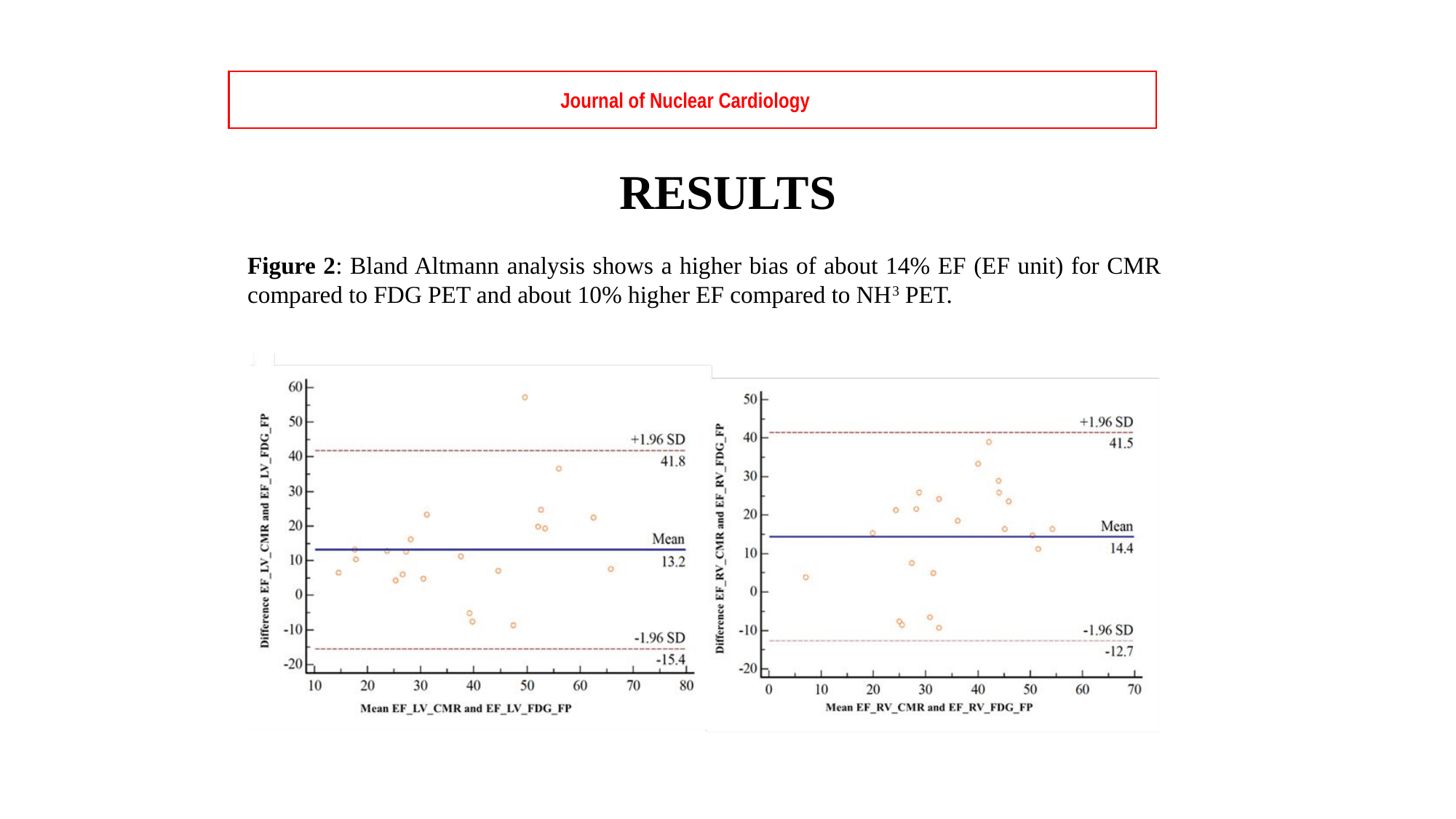

Journal of Nuclear Cardiology
RESULTS
Figure 2: Bland Altmann analysis shows a higher bias of about 14% EF (EF unit) for CMR compared to FDG PET and about 10% higher EF compared to NH3 PET.

## Slide 7
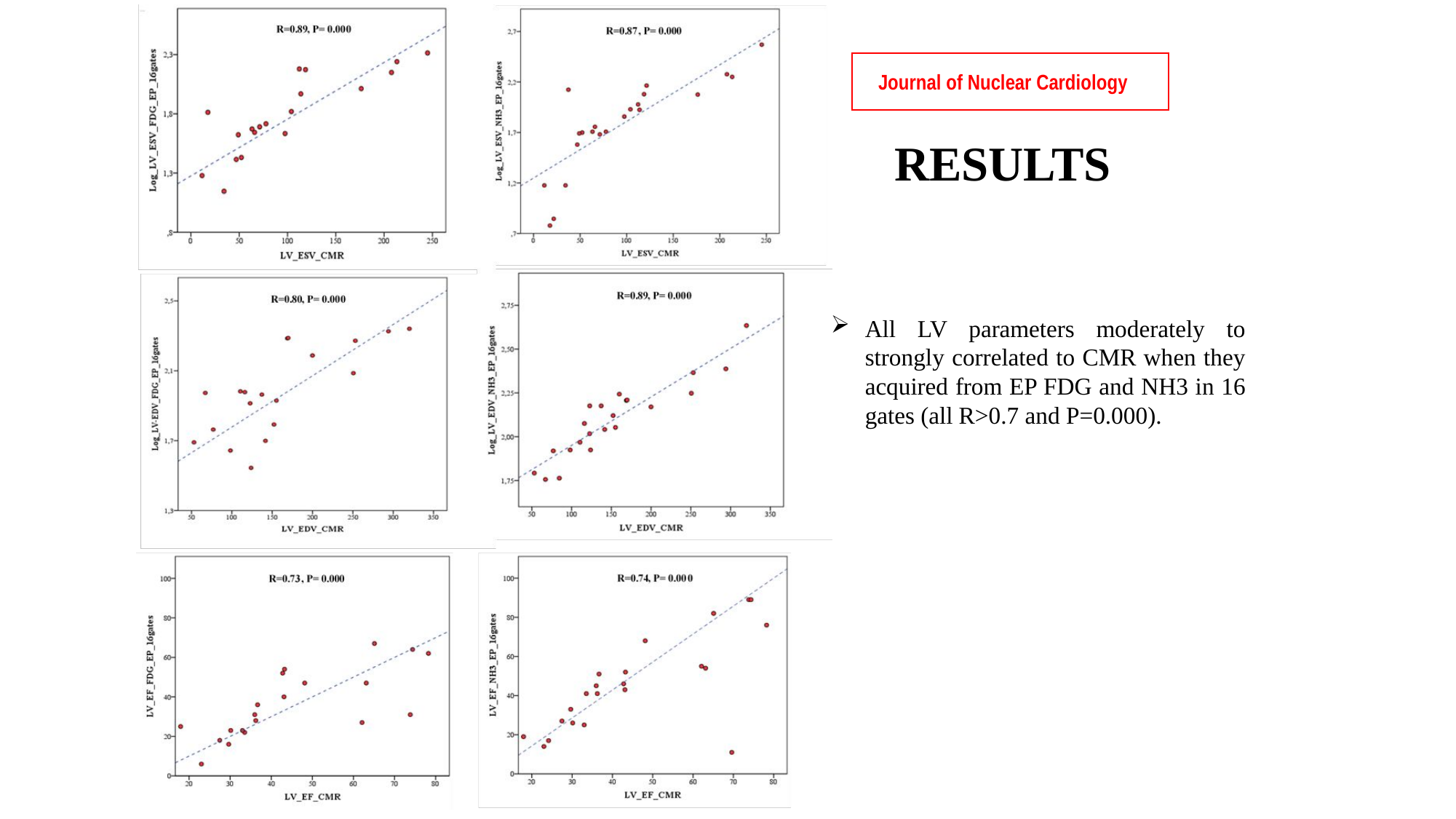

Journal of Nuclear Cardiology
RESULTS
All LV parameters moderately to strongly correlated to CMR when they acquired from EP FDG and NH3 in 16 gates (all R>0.7 and P=0.000).

## Slide 8
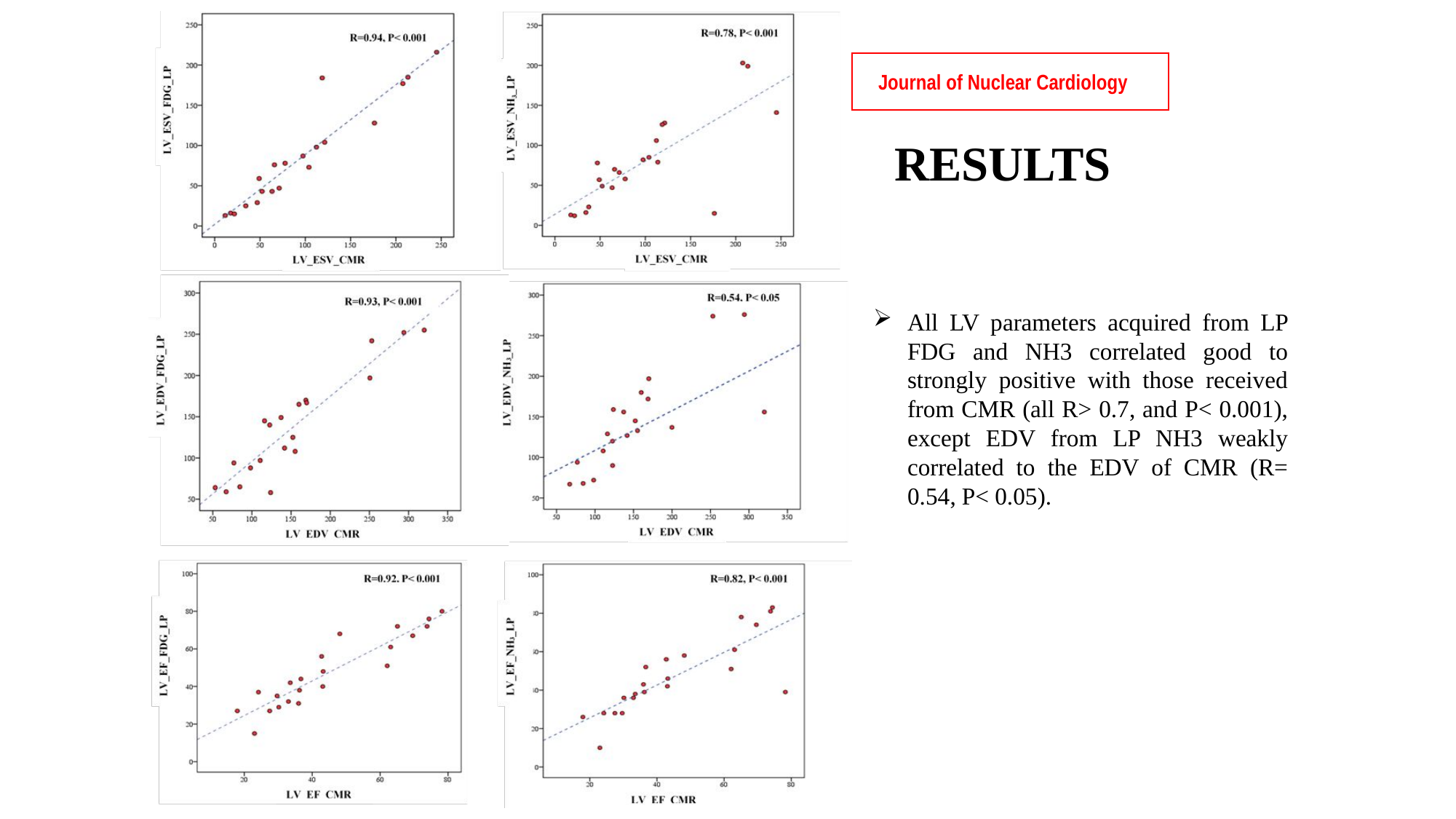

Journal of Nuclear Cardiology
RESULTS
All LV parameters acquired from LP FDG and NH3 correlated good to strongly positive with those received from CMR (all R> 0.7, and P< 0.001), except EDV from LP NH3 weakly correlated to the EDV of CMR (R= 0.54, P< 0.05).

## Slide 9
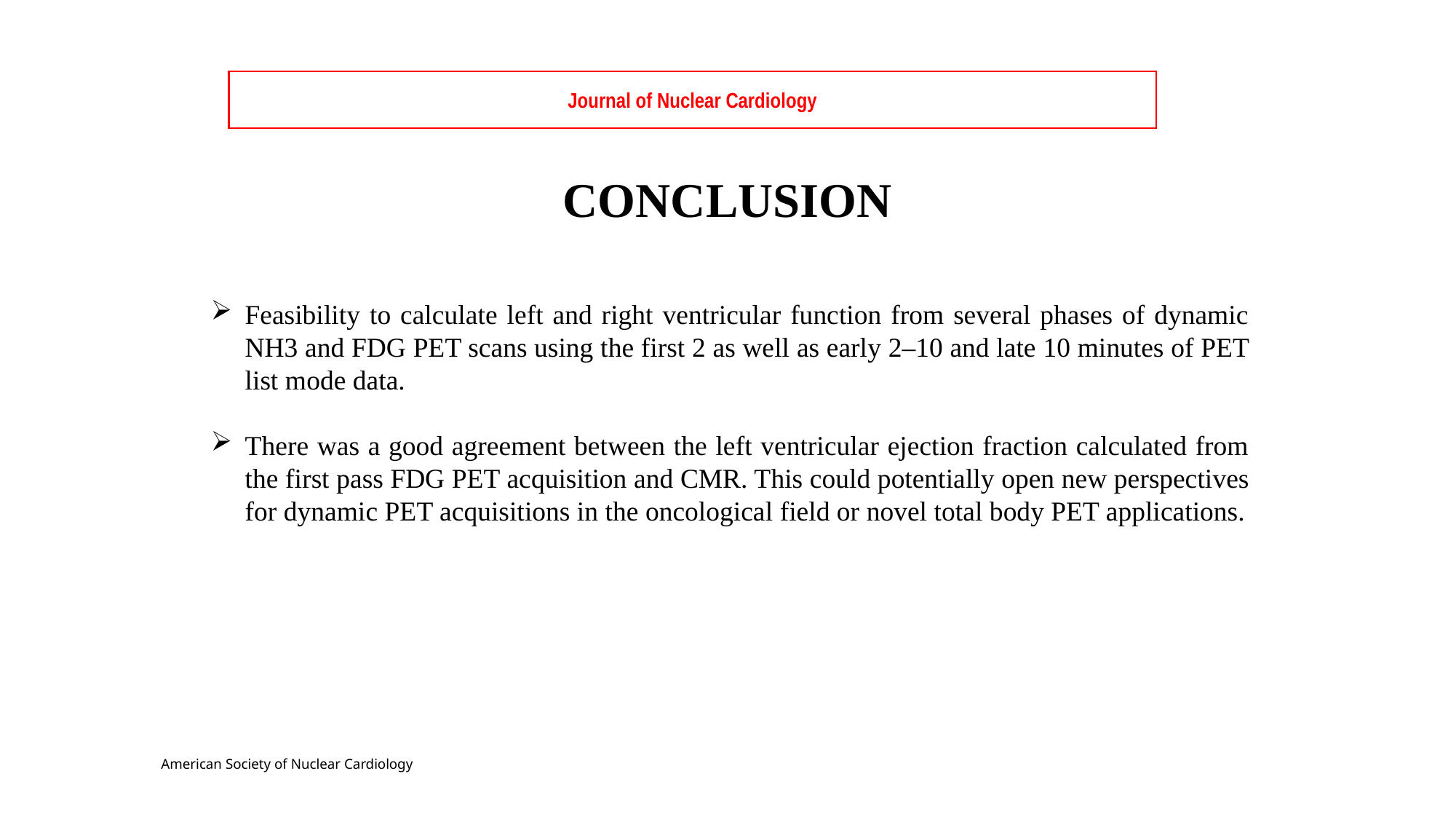

Journal of Nuclear Cardiology
CONCLUSION
Feasibility to calculate left and right ventricular function from several phases of dynamic NH3 and FDG PET scans using the first 2 as well as early 2–10 and late 10 minutes of PET list mode data.
There was a good agreement between the left ventricular ejection fraction calculated from the first pass FDG PET acquisition and CMR. This could potentially open new perspectives for dynamic PET acquisitions in the oncological field or novel total body PET applications.
American Society of Nuclear Cardiology

## Slide 10
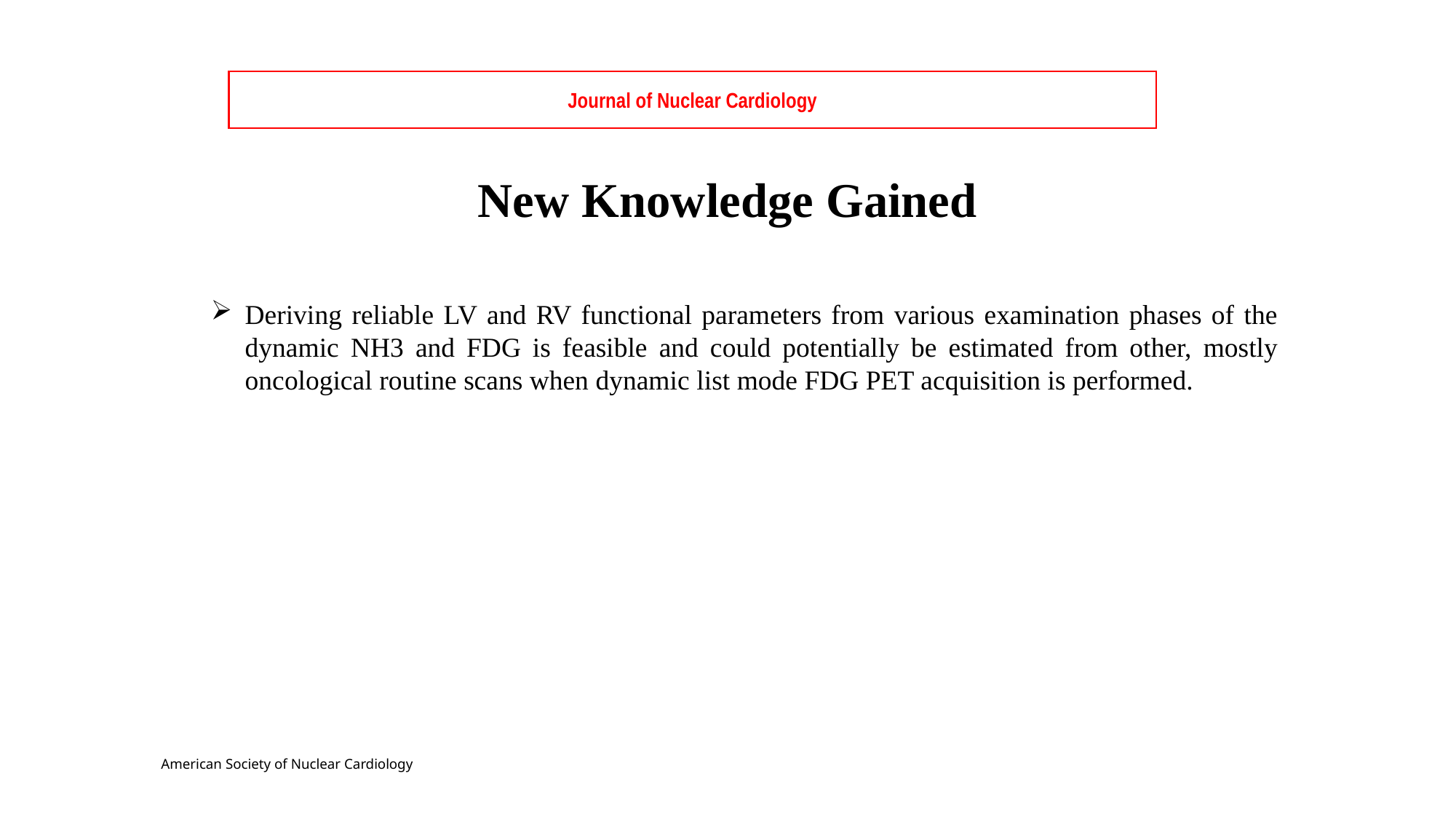

Journal of Nuclear Cardiology
New Knowledge Gained
Deriving reliable LV and RV functional parameters from various examination phases of the dynamic NH3 and FDG is feasible and could potentially be estimated from other, mostly oncological routine scans when dynamic list mode FDG PET acquisition is performed.
American Society of Nuclear Cardiology
